# Autoimmune phenotype with type I interferon signature in two brothers with ADA2 deficiency carrying a novel *CECR1* mutation

**DOI:** 10.1186/s12969-017-0193-x

**Published:** 2017-08-22

**Authors:** Andrea Skrabl-Baumgartner, Barbara Plecko, Wolfgang M. Schmidt, Nadja König, Michael Hershfield, Ursula Gruber-Sedlmayr, Min Ae Lee-Kirsch

**Affiliations:** 1 Department of Pediatrics and Adolescent Medicine, Medical University Graz, Auenbruggerplatz 34/2, 8036 Graz, Austria; 2 Division of Child Neurology, University Children’s Hospital, University of Zurich, Zurich, Switzerland; 3 Neuromuscular Research Department, Center for Anatomy and Cell Biology, Medical University of Vienna, Vienna, Austria; 4 Department of Pediatrics, Medical Faculty, Technical University Dresden, Dresden, Germany; 5 Department of Medicine and Biochemistry, Duke University School of Medicine, Durham, NC USA

**Keywords:** Adenosine deaminase 2, *CECR1*, Systemic lupus erythematosus, Hypergammaglobulinaemia, Antinuclear antibodies, Interferon signature

## Abstract

**Background:**

Loss-of-function *CECR1* mutations cause polyarteritis nodosa (PAN) with childhood onset, an autoinflammatory disorder without significant signs of autoimmunity. Herein we describe the unusual presentation of an autoimmune phenotype with constitutive type I interferon activation in siblings with adenosine deaminase 2 (ADA2) deficiency.

**Case presentation:**

We describe two siblings with early-onset recurrent strokes, arthritis, oral ulcers, discoid rash, peripheral vascular occlusive disease and high antinuclear antibody titers. Assessment of interferon signatures in blood revealed constitutive type I interferon activation. Aicardi-Goutières syndrome (AGS) was suspected, but no mutation in the known AGS genes were detected. Whole exome sequencing identified compound heterozygosity for a known and a novel mutation in the *CECR1* gene. Functional consequences of the mutations were demonstrated by marked reduction in ADA2 catalytic activity.

**Conclusions:**

Our findings demonstrate that ADA2 deficiency can cause an unusual autoimmune phenotype extending the phenotypic spectrum of PAN. Constitutive interferon I activation in patient blood suggests a possible role of type I interferon in disease pathogenesis which may have therapeutic implications.

## Background

Deficiency of adenosine deaminase 2 (ADA2) is a rare systemic vascular inflammatory disorder, also known as polyarteritis nodosa with childhood-onset (PAN; OMIM 615688). PAN is caused by autosomal recessive loss-of-function mutations in the *CECR1* gene encoding adenosine deaminase 2 [1, 2]. Since the first description of familial PAN in 2014, 19 pathogenic mutations have been described. The disorder shows a variable age of onset, although most patients present with symptoms in the first decade [3, 4]. The main clinical findings include early-onset recurrent strokes, livedo reticularis and recurrent fever. Organ involvement, course and severity vary largely, even among affected individuals of the same family, ranging from mild skin disease to devastating systemic disease. Whereas cytopenia and hypogammaglobulinemia associated with varying degrees of immunodeficiency have commonly been described in ADA2-deficient patients [1, 4–7], signs of autoimmunity have only rarely been recognized. Although the pathogenesis of the disease is currently not entirely understood, markers of cellular immunity and macrophage activation have shown to be increased, suggesting a role of ADA2 in immune responses. Here we describe two siblings who presented with early-onset cerebrovascular disease and autoimmune features caused by ADA2 deficiency.

## Case presentation

**Fig. 1:**
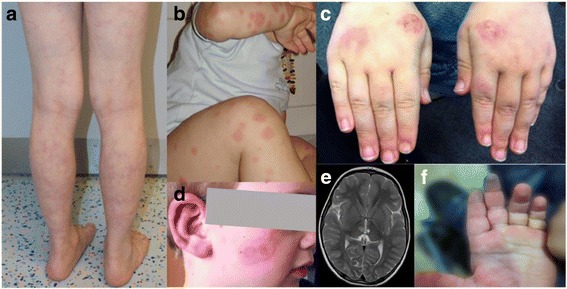
Clinical findings of the patients. **a** Livedo reticularis of patient 1. **b**, **c**, **d** Erythematous skin lesions of patient 2, which appear as discoid plaques or annular papules. **e** T2-weighted magnetic resonance image of the brain of patient 2 showing a recent ischemic lesion in the *right* thalamus and an older lesion in the *left* thalamus. **f** Blue finger syndrome due to vascular occlusion in patient 2

Patient 1 was born after an uneventful pregnancy and delivery to healthy nonconsanguineous Caucasian parents. At 22 months of age, he presented with recurrent episodes of unexplained fever, mouth ulcers and abdominal pain with intermittently elevated C-reactive protein (CRP). At age 3 years, he developed supranuclear ophthalmoplegia and right oculomotor nerve palsy. Cerebral magnetic resonance imaging (MRI) and cerebrospinal fluid (CSF) were unremarkable then. CRP and ESR were markedly increased, as were serum IL-6 and s-IL2R. Within 2 weeks, neurological symptoms resolved completely, but febrile episodes, abdominal pain and mouth ulcers recurred. In addition, he developed livedo reticularis and arthralgia (Fig. 1a). Follow-up blood tests revealed chronic inflammation. Antinuclear antibodies (ANA) were positive (1:320) with a homogenous pattern, while antibodies to extractable nuclear antigens (ENA), anti-double-stranded DNA (dsDNA)-, anticardiolipin (aCL)- and anti-beta2-glycoprotein (anti-ß2GP)-antibodies were negative, as were anti-neutrophilic antibodies (ANCA). At age 5 years, he presented with mild right-sided hemiparesis. Cerebral MRI revealed a 5 mm signal alteration in the brainstem consistent with ischemia. MR angiography was without pathologic findings. An underlying coagulopathy and infections were excluded. ANA titer was increased to 1:640. Therapy with heparin was started, followed by low-dose acetylsalicylic acid (ASA). Complete neurological remission was achieved within 1 week. From age 7 to 9 years, the patient experienced recurrent attacks of vertigo and headaches, episodes of abdominal pain and recurrent mouth ulcers. He continued to have prominent livedo and arthralgia of small joints and knees accompanied by swelling and morning stiffness. At 10 years of age, he suffered an episode of severe ataxia, nystagmus, and diplopia. Laboratory findings included increased markers of inflammation, hypergammaglobulinemia and an ANA titer of 1:1280. Lupus-anticoagulant was transiently positive, while complement factors C3 and C4 were repeatedly within normal limits. A skin biopsy revealed unspecific teleangiectasia of small vessels with sparse inflammatory infiltrates. At the last follow-up visit ANA titer was 1:1280 and anti-nucleosome antibodies were positive for the first time.

Patient 2 is the younger brother of patient 1. He presented at 2.5 years of age with acute right-sided hemiparesis, accompanied by annular violaceous skin lesions on both calves and mouth ulcers. Cerebral MRI showed an ischemic lesion in the left thalamus (Fig. 1e). Inflammatory markers as well as cytokines IL-6 and s-IL2R were increased and ANA were positive (1:1280) with a homogenous pattern; anti-ENA-, anti-ds DNA-antibodies, anticardiolipin (aCL)- and anti-beta2-glycoprotein (anti-ß2GP)-antibodies as well as ANCA were negative, and circulating immune complexes were unremarkable, as were levels of C3, C4 and CH50. Suspecting a systemic autoimmune disease with neuroinflammation, therapy with oral steroids at a dose of 2 mg/kg and low-dose ASA was started with complete recovery of neurologic symptoms within several days. Four weeks later, while tapering steroids, the patient developed a left-sided facial palsy. A repeated MRI revealed an ischemic lesion in the right thalamus; CRP and ESR were markedly elevated. Steroids were increased to 2 mg/kg and neurological symptoms again resolved within a few days. Steroids thereafter were tapered off over 3 weeks. Two months later, the patient complained of recurrent abdominal pain, and mouth ulcers accompanied by relapsing fever and fatigue. Ultrasound examination of abdomen and urinary tract, echocardiography and chest-x-rays were without pathologic findings, as were liver and renal function tests. Despite elevated calprotectin in stool (480 μg/g), endoscopy of the gastrointestinal tract was unremarkable. Thereafter, he presented with annular lesions on the face and sun-induced eczematous lesions on both hands (Fig. 1c-d). At age 3.5 years, Raynaud’s phenomenon, pain and morning stiffness affecting several large and small joints were noted along with livedo racemosa. At follow-up visits, he presented with arthritis of the left metacarpophalangeal (MCP) joints. An ultrasound revealed nonerosive synovitis. He continued to show chronic inflammation, hypergammaglobulinemia and an ANA titer of 1:2560. Arthritis of MCP joints persisted and he developed a blue finger syndrome of the third digit (Fig. 1f). Pulse oscillography revealed a critically limited perfusion due to occlusion of small digital arteries. Therapy with nifedipine, oral steroids and azathioprine (AZA) was initiated and low-dose ASA continued. Under this regimen, arthritis, abdominal pain as well as febrile episodes significantly improved. After 3 years of treatment, AZA was stopped. Continuing low-dose ASA, the patient showed mildly impaired fine motor skills and marked livedo accompanied by slightly increased markers of inflammation as well as an ANA titer of 1:1280.

**Fig. 2:**
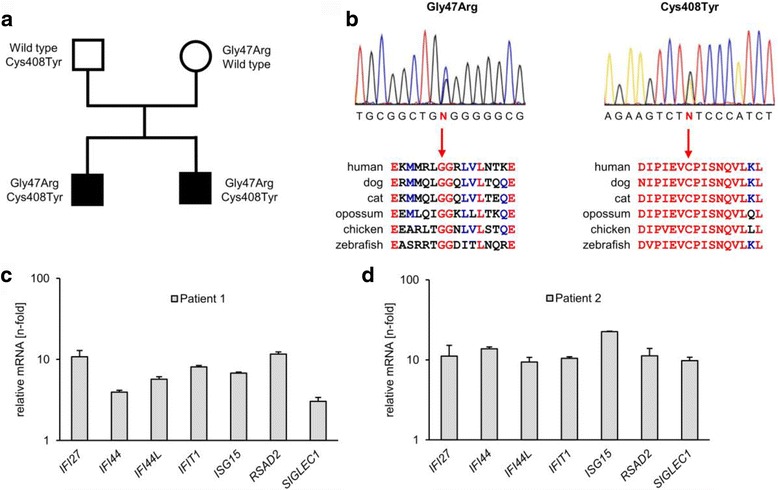
Genetic and molecular findings. **a** Pedigree of the family with ADA2 deficiency. *Solid symbols* indicate affected persons, *open symbols* unaffected relatives, *squares* male persons, *circles* female persons. The amino acid changes are indicated next to the symbols. **b** The *upper panel* shows electropherograms with the two heterozygous *CECR1* mutations, c.139G>C (p.Gly47Arg) and c.1223G>A (p-Cys408Tyr), identified in both siblings. The *lower panels* show alignments of ADA2 protein sequences from different species. The positions of the altered amino acid residues Gly47 and Cys408 are marked by *arrows*. Highly conserved regions are marked in *red* and less conserved regions are indicated in *blue*. **﻿﻿c**, **d**﻿ ﻿Expression of interferon-stimulated genes, *IFI27*, *IFI44*, *IFI44L*, *IFIT1*, *ISG15*, *RSAD2* and *SIGLEC1* in peripheral blood. Shown is the fold-change of the respective mRNA relative to the mean expression of 10 healthy controls

Since early-onset inflammatory systemic vascular disease affecting the brain and skin is commonly observed in patients with type I interferonopathies, both patients were investigated for signs of an interferon signature in blood [8]. Quantitative RT-PCR of peripheral blood cells revealed enhanced expression of interferon-stimulated genes demonstrating constitutive type I IFN activation (Fig. 2c-d). Based on these clinical findings, the patients were initially suspected of Aicardi-Goutières syndrome (AGS). However, no mutation in any of the seven known AGS genes were detected.

Whole exome sequencing of patient 1 was therefore performed to identify the disease-causing mutation. Variant analysis and filtering assuming a recessive mode of inheritance revealed two missense mutations within the *CECR1* gene, c.139G>C (p.Gly47Arg) located in exon 1 and c.1223G>A (p.Cys408Tyr) in exon 7 (Fig. 2a, b). Targeted Sanger sequencing confirmed the presence of both mutations in patient 2, the affected brother. Furthermore, both parents were found to carry only one of the two mutations, in line with compound heterozygosity for both mutations in the affected sons (Fig. 2a-b). While the first mutation (p.Gly47Arg) has been described as the most common pathogenic variant in ADA2-deficiency [1, 2], the second mutation (p.Cys408Tyr) has previously not been associated with a disease phenotype. This variant is listed in the ENSEMBL database as variant rs751280698 and was found only once in a single individual in the heterozygous state within the ExAc data base corresponding to an allele frequency of 8.23 × 10^−6^, which is compatible with a rare disease-causing recessive variant. p.Cys408Tyr causes the exchange of a cysteine residue to tyrosine within the adenosine/adenosine monophosphate deaminase domain and is predicted to be “probably damaging” by PolyPhen (score 0.998) and “deleterious” by SIFT (score 0). Alignment of the ADA2 protein sequences by hierarchical clustering revealed a high degree of conservation of amino acid residue Cys408 of the ADA2 protein across multiple species including zebrafish (Fig. 2b).

To further explore the functional consequences of the *CECR1* mutations, ADA2 enzyme was measured in patients and parents as well as in healthy controls by the dried plasma spot method as previously described [1]. A marked reduction in enzyme activity was noted in both patients (patient 1, 0.6 mU/g; patient 2, 0.04 mU/g) compared to healthy controls (control 1, 81.3 mU/g; control 2, 120.0 mU/g). The level of enzyme activity was below the average enzyme activity of 29 ADA2-deficient patients of 4.7 ± 4.2 mU/g (range, 0.6–17.2 mU/g), confirming that both, p.Gly47Arg and p.Cyc408Tyr act as loss-of-function mutations.

After ADA2 deficiency was diagnosed, ASA was stopped in both patients and treatment with anti-TNF agent was recommended.

## Discussion

In this report, we describe two siblings with early-onset recurrent strokes, systemic inflammation, erythematous skin lesions, livedo reticularis as well as arthritis accompanied by high ANA titers and ascribe this inflammatory phenotype to biallelic *CECR1* mutations. While one of the identified mutation p.Gly47Arg represents the most common mutation found in patients with PAN, the second mutation p.Cys408Tyr is novel and affects a highly conserved residue within the catalytic domain of the ADA2 enzyme. Functional testing confirmed a marked reduction of ADA2 enzyme activity in agreement with a loss of function. Whereas clinical features of vasculitis and mild immunodeficiency due to cytopenia and hypogammaglobulinemia are commonly observed in patients with ADA2 deficiency, autoimmunity has only rarely been described [1, 4–7].Thus, low ANA titers have been reported in 9 out of 45 ADA2-deficient patients [3], while hypergammaglobulinemia has been reported only in two siblings with additional IL-17RA deficiency [9]. An autoimmune phenotype with discoid skin lesions with partial photosensitivity, oral ulcerations, non-erosive arthritis, abdominal pain as well as neurological signs due to cerebrovascular disease, together with the constantly high ANA titers, transiently elevated lupus anticoagulants, anti-nucleosome antibodies, hypergammaglobulinemia and anemia, has not been described previously. These findings are overlapping with features seen in patients with systemic lupus erythematosus (SLE) and other type I interferon-driven disorders [10]. Interestingly, both patients exhibited a repeatedly high expression of interferon-stimulated genes in blood indicating constitutive type I interferon activation. Such an interferon signature is typically seen in patients with SLE or Mendelian forms of type I interferon-driven disorders such as chilblain lupus or Aicardi-Goutières syndrome [11, 12]. As upregulation of IFN stimulated genes has recently been observed in 4 patients with ADA2 deficiency, our data suggest a contributing pathogenetic role of systemic type I interferon activation in ADA2 deficiency [13, 14]. Further studies are necessary to evaluate the potential efficacy of JAK inhibition, besides the proven therapeutic effect of TNF inhibitors, for this entity.

## Conclusion

Our findings demonstrate that ADA2 deficiency can cause an autoimmune phenotype, extending the phenotypic spectrum PAN. This implies that ADA2 deficiency should be considered in the differential diagnosis of pediatric patients presenting with early-onset autoimmune ﻿phenomena﻿ as this ﻿knowledge will be important for clinical decisions with respect ﻿to specific treatment options. Constitutive type I interferon activation in patient blood suggests a possible role of type I interferon in disease pathogenesis, although the underlying molecular mechanisms remain to be investigated.

## Abbreviations

ADA2: Adenosine deaminase 2
ANA: Antinuclear antibodies
*CECR1*: Cat eye syndrome chromosome region, candidate 1
PAN: Polyarteritis nodosa
SLE: Systemic lupus erythematosus
